# Eosinophilic Variant of Chromophobe Renal Cell Carcinoma During Pregnancy: A Multidisciplinary Approach and Successful Management in a Tertiary Hospital in Mexico

**DOI:** 10.7759/cureus.44955

**Published:** 2023-09-09

**Authors:** Daniel A Meza-Martinez, Jose H Hernandez-Hernandez, Brando J Fematt-Rodriguez, Miguel A Meza-Martinez, Helio Rios-Rosales

**Affiliations:** 1 General Surgery, Instituto Mexicano del Seguro Social, Hospital General de Zona No. 33, Monterrey, MEX; 2 Urology, Instituto Mexicano del Seguro Social, Unidad Médica de Alta Especialidad, Hospital de Especialidades No. 2 Luis Donaldo Colosio Murrieta, Obregón, MEX

**Keywords:** non-clear cell renal carcinoma, second trimester pregnancy, pregnancy surveillance, renal neoplasm, laproscopic urology, unexplained recurrent pregnancy loss, high-risk pregnancy, laparoscopic radical nephrectomy

## Abstract

Renal cell carcinoma (RCC) is rarely diagnosed during pregnancy and its management represents a challenge as it necessitates considerations for the well-being of both the mother and the developing fetus. Diagnosis can be challenging and is often an incidental finding during routine imaging, which can lead to difficult decision-making. The choice of the ideal imaging study in these cases is a matter of debate. When the tumor is detected at an early stage, radical nephrectomy is indicated. However, there is still controversy regarding whether it should be performed conventionally or laparoscopically, as both techniques have their risks and benefits. In this context, our primary objective was to provide adequate surgical treatment for the patient, while safeguarding fetal health. Here, we present a patient with a history of recurrent miscarriages, in whom a renal tumor was incidentally diagnosed during pregnancy. Adding to the uniqueness of this case, the patient was diagnosed with an eosinophilic variant of chromophobe RCC through histopathological analysis. Our aim is to highlight the controversies surrounding diagnostic and treatment methodologies and to present the surgical techniques employed in this unique situation. This case underscores the importance and need for a multidisciplinary approach, which, in our instance, resulted in favorable outcomes for both maternal and neonatal health.

## Introduction

Urological cancers are a highly uncommon phenomenon during gestation, with an incidence rate of approximately 13 cases per 1,000,000 pregnancies and renal cell carcinoma (RCC) emerging as the most frequently reported renal tumor in pregnancy, followed by angiomyolipoma and blastoma [1]. It presents with symptoms, such as a flank mass, flank pain, hematuria, fever, and hypertension [2]. RCC may be detected as an incidental finding during routine ultrasonography. Determining the most suitable timing for surgery while considering its effects on both maternal and fetal well-being holds significant importance [3].

When a renal tumor is detected in the first trimester, it is essential to address its integral management. The dilemma in treatment should be communicated to the patient, emphasizing the risks to pregnancy outcomes and discussing the option of pregnancy termination. When the renal mass is diagnosed in the second or third trimester, surgical intervention can be conducted. By contrast, postponing surgery after birth is considered a viable option by some authors as a favorable approach, although there are no robust data to support treatment decisions in these cases [3,4]. Within this context, recent studies have emphasized the absence of a consensus in managing such scenarios [1].

This is the case of a 36-year-old female patient with a history of spontaneous abortions. An incidental renal mass was discovered during a routine ultrasonographic evaluation, which was subsequently diagnosed as a rare variant of RCC during pregnancy. This case underscores the importance of a multidisciplinary approach, with a primary focus on ensuring optimal management for both the mother and her fetus.

## Case presentation

We present a case of a 36-year-old female patient with a past obstetric history of G3P0A2, which includes two intrauterine curettages in 2016 and early 2018 due to spontaneous abortions during the first trimester. Her gynecological and obstetric history revealed that she was diagnosed as pregnant at six weeks of gestation due to a delayed period, without exhibiting any other pregnancy-related symptoms, and at that time, her last menstrual period date was May 26, 2019. After the second curettage, a routine pelvic and abdominal ultrasound was performed, which demonstrated a right renal cyst without any other specifications. According to the patient's account, she was informed that her renal cyst was congenital in nature and did not require continuous follow-up.

In June 2019, she was referred to our urology department by her gynecologist due to her history of intrauterine curettages following spontaneous miscarriages. During the evaluation, the obstetrician requested a series of tests, including an abdominal ultrasound, which once again revealed the previously mentioned renal mass. On this occasion, the tumor exhibited characteristics indicative of malignancy, as rapid growth was evidenced when compared to previous imaging studies. Therefore, the radiologist recommended complementing these findings with a contrast-enhanced computed tomography (CT), despite her pregnancy status.

**Figure 1:**
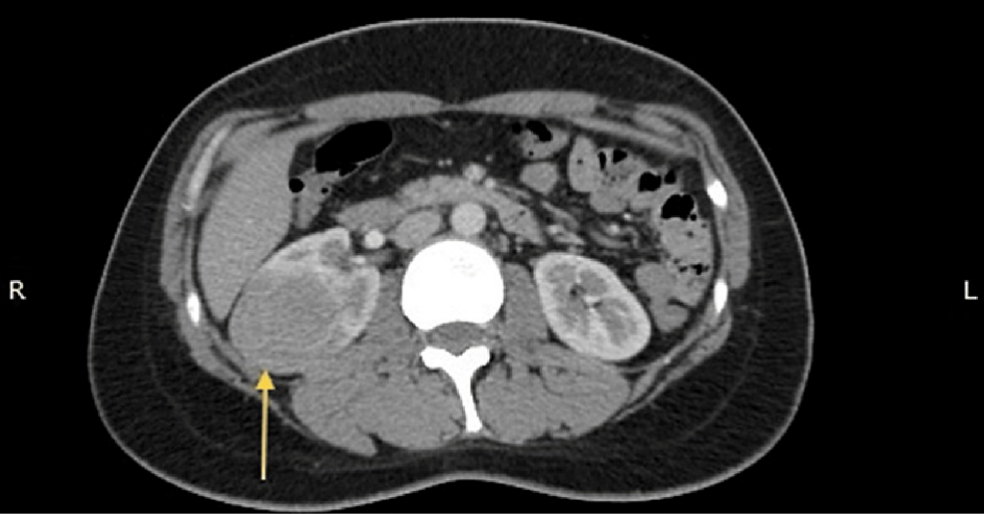
CT scan in the axial plane that shows a mass (arrow) located on the mid-portion of the right kidney. CT: computed tomography

**Figure 2:**
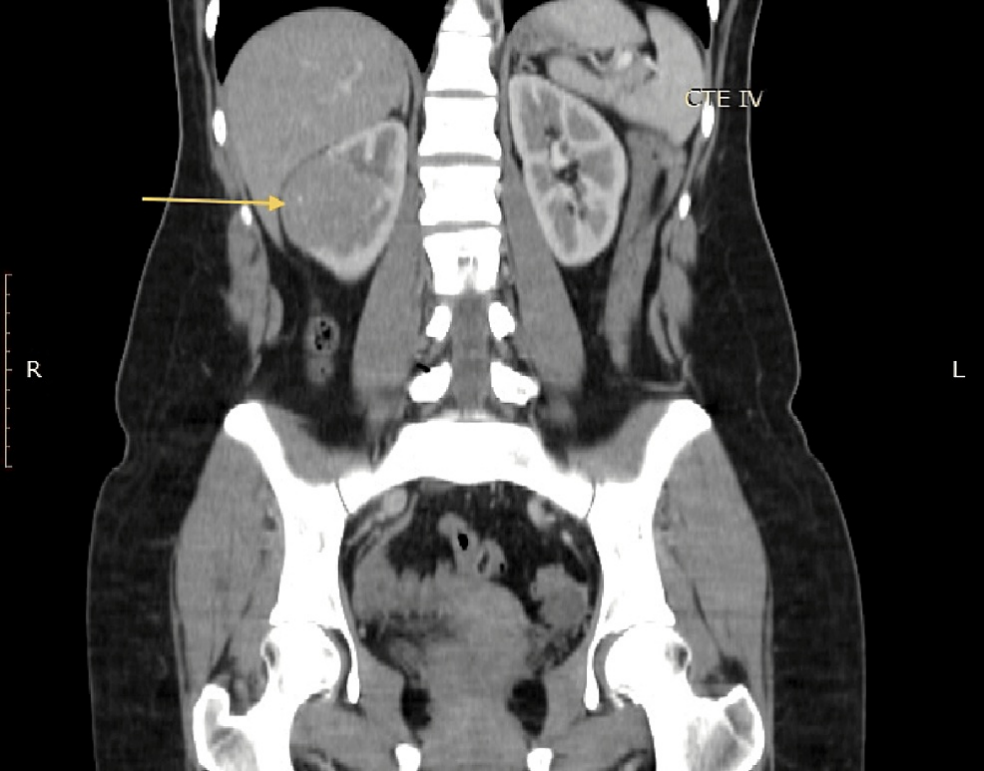
CT scan in a coronal plain that demonstrates the same renal tumor (arrow), which showed enhancement after contrast administration. CT: computed tomography

The CT scan was performed on June 15, 2019 and revealed a heterogeneous mass in the middle portion of the right kidney. The tumor measured approximately 4.5 x 4.2 x 4.6 cm (Figure 1) and showed enhancement after contrast administration, increasing from 32 to 64 Hounsfield units (HU) (Figure 2). Regional lymph nodes showed no alterations, and the rest of the study was unremarkable.

As a result of the previous findings, she was referred to the tertiary level of care with a diagnosis of a right renal tumor classified as T1bN0M0 and a pregnancy at 8.3 weeks of gestation. During her initial visit to our urologic service, a comprehensive medical history and examination were conducted. The patient remained entirely asymptomatic, and no noteworthy findings were documented during the physical examination. As a part of our initial approach, we conducted routine laboratory tests, all of which returned within normal ranges.

Given the complexity of the case, we convened a multidisciplinary session involving the expertise of the oncology, gynecology-obstetrics, and radiology departments to determine the optimal management approach. At 12 weeks of gestation, a consultation with the perinatology department was deemed necessary, and a transvaginal ultrasound confirmed fetal viability, with no other abnormalities noted in the medical records. Considering that the cornerstone of her treatment was surgical, after a thorough discussion, we concluded that the optimal timing for the intervention was the second trimester of pregnancy. We also agreed that deferring the procedure until the end of gestation was unnecessary. Similarly, there was no indication for early termination of the pregnancy given the absence of metastatic disease at the time. Regarding the choice of surgical technique, we deemed a laparoscopic approach as the most suitable for this case.

**Figure 3:**
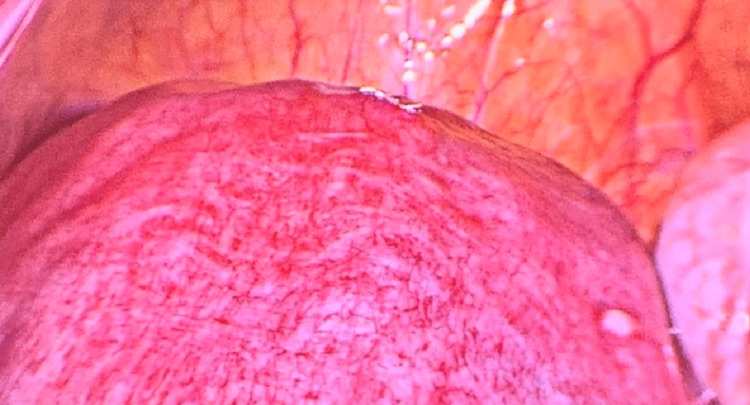
Enlarged uterus observed during diagnostic laparoscopy, which correlated with the patient's weeks of gestation.

Taking all of the above into account, the patient underwent surgery during the 18th week of gestation, as our main goal was the complete resection of the tumor. One of the key technical aspects of the surgery highlighted the positioning of the patient in the left lateral decubitus. A right paraumbilical incision was made using the Hasson technique, through which a 12-mm port was inserted and used to establish the pneumoperitoneum. Diagnostic laparoscopy was performed, observing an enlarged uterus that correlated with the patient’s weeks of gestation (Figure 3).

**Figure 4:**
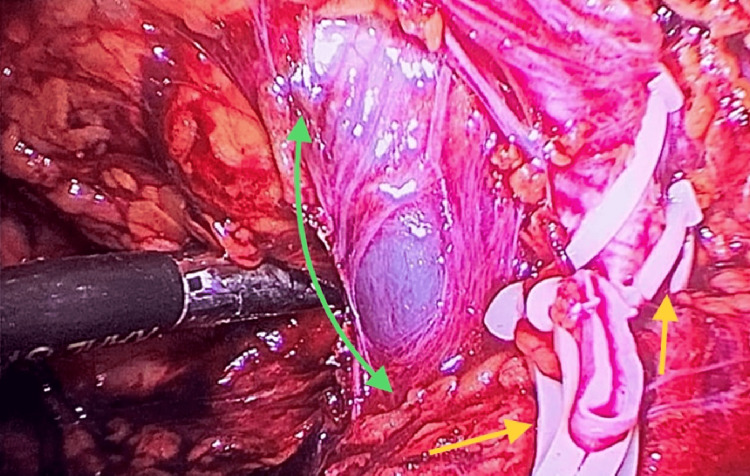
Laparoscopic image of renal vessels, showing an arterial anatomical variant depicted by the presence of two ligated and cut renal arteries (yellow arrows). In addition, a single renal vein is observed, dissected along its course with no evidence of tumor invasion (green arrow).

We then placed the remaining ports under direct visualization: a 12 mm port was placed in the right subcostal region for surgical specimen mobilization, another 5 mm port in the subxiphoid area for hepatic retraction, and an additional 5 mm port in the right flank, which was utilized during the assistance and mobilization of the surgical specimen. We initially mobilized the ascending colon, incising the Toldt's fascia and gained access to the retroperitoneum. We then identified the ureter and the vessels of the renal hilum, placing two proximal ligatures and one distal ligature on each of its components. The renal hilum was composed of two renal arteries and a renal vein, which were subsequently transected using endoscopic scissors (Figure 4).

**Figure 5:**
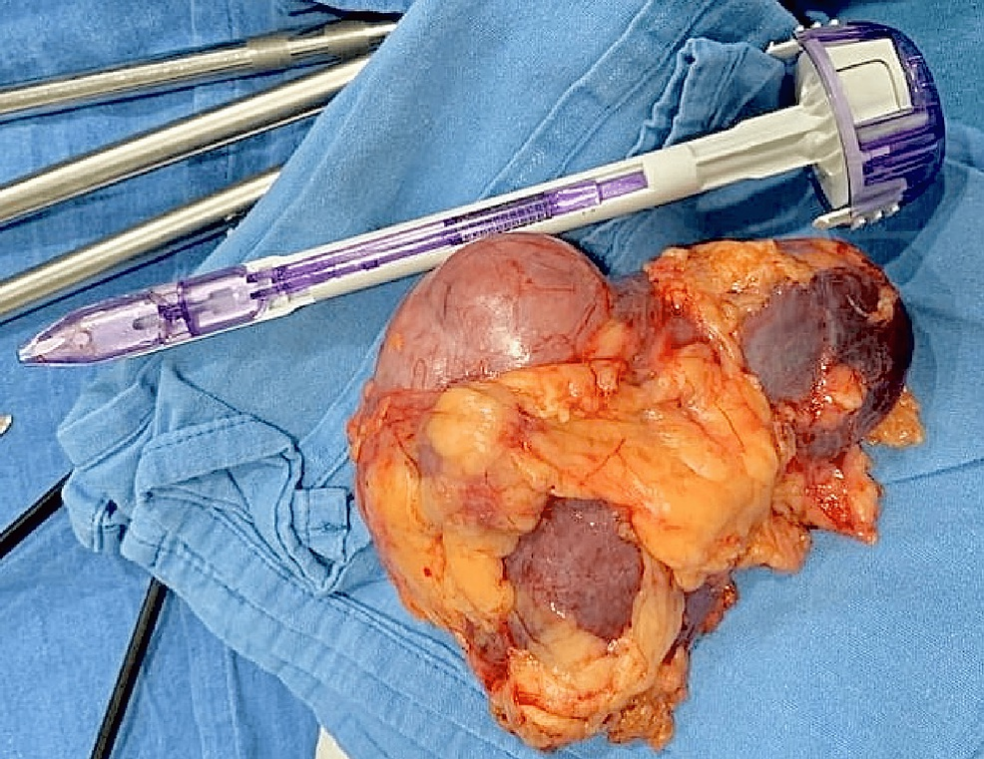
Surgical specimen that shows a right renal kidney with a well-defined tumor located at its mid pole. Trocar used as a reference for size comparison.

Subsequently, we caudally dissected the ureter until its ligation and division. We proceeded with the dissection of the perirenal adipose tissue, successfully detaching the surgical specimen. Finally, we extracted the right kidney, carefully inspecting the cavity for any sites of residual bleeding. The remaining steps of the procedure were executed in a standard manner without encountering any complications, resulting in a total surgical duration of 180 minutes. Considering all the information mentioned earlier, the procedure carried out was a right laparoscopic radical nephrectomy. The surgical findings revealed a right kidney measuring 12 x 7 x 6 cm with a tumor located at its mid pole with no involvement of adjacent structures (Figure 5). The ureter appeared macroscopically normal. An anatomical variant was noted by the presence of two renal arteries. The renal vein exhibited no alterations. Regional lymph nodes displayed no abnormalities.

There were no surgical, obstetric, or anesthetic complications. The patient did not present symptoms suggestive of threatened abortion, and no tocolytic agents were administered. She recovered appropriately and was discharged the following day after fetal viability was confirmed.

**Figure 6:**
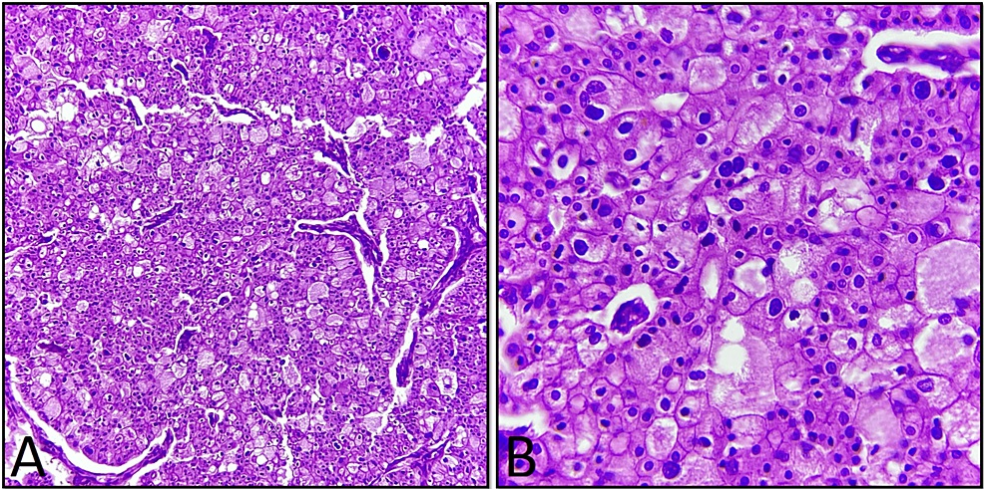
Histopathological images of the excised tumor. A. Histological picture showing chromophobe neoplastic cells with eosinophilic cytoplasm (H&E 10x). B. Microscopic image that demonstrates small, binucleate cells, a common finding in eosinophilic variant of chromophobe RCC (H&E 40x). H&E: hematoxylin and eosin

The specimen was sent to the pathology department for analysis. The pathology report described a kidney measuring 12 x 7 x 6 cm, covered by an intact capsule and containing scant adipose tissue. No anomalies were noted upon examination of the renal hilum. Upon gross examination, a well-defined tumor measuring 8 x 6 x 6 cm, situated at the mid pole of the kidney, was identified. It displayed a white-grayish and friable appearance. The remaining renal parenchyma exhibited a proper cortex-to-medulla ratio, displaying a reddish-brown and congestive appearance. Histopathological examination confirmed the diagnosis of an eosinophilic variant of chromophobe RCC (Figure 6), which had invaded but not ruptured the renal capsule. Surgical margins were reported as tumor-free, and no vascular or lymphatic involvement was observed.

Regarding follow-up, the patient attended her prenatal appointments promptly and did not experience any threatened miscarriage signs or symptoms during her pregnancy. Her medical record shows that the rest of the gestation was conducted normally. The pregnancy was terminated via cesarean section at 39 weeks as per the patient's request. The newborn exhibited no malformations, weighed 3,450 grams, and received Apgar scores of 8 and 9 at one and five minutes, respectively. She decided to continue her oncological follow-up at a private hospital in her natal city due to pandemic related issues. Her medical record reports that she undergoes imaging studies every six months, and as of December 2022, there was no evidence of tumor recurrence. From our end, the patient attends yearly appointments, and throughout the current year, no postoperative complications or evidence of recurrence have been detected.

## Discussion

RCC presenting during gestation is an exceedingly rare phenomenon, with approximately 100 reported cases in the literature. However, renal cancer remains the most commonly diagnosed urologic tumor in pregnancy. Elevated blood pressure, estrogen levels during gestation, and the occurrence of RCC in pregnant women have been linked as risk factors. During the initial assessment, a substantial portion of pregnant patients exhibit no symptoms, and renal tumors are typically incidentally detected through routine ultrasound examinations [5]. These observations closely align with the presentation of our case. Nonetheless, upon reviewing recent literature, we did not uncover any evidence indicating that RCC serves as a potential cause of recurrent pregnancy loss. Hence, we believe that the miscarriages encountered by this patient were probably caused by an alternative underlying factor.

Recent literature reveals that typical RCC symptoms, when present, encompass pain (50%), hematuria (47%), and hypertension (18%), while the classical triad of back pain, mass, and hematuria is observed in 26% of pregnant patients. The typical symptoms of RCC differ when presenting in a pregnant patient due to the potential overlap between them, such as abdominal pain, abdominal distension, and increased abdominal circumference. The aforementioned factors can mask the clinical picture and lead to diagnostic delays [6].

Although our patient exhibited no symptoms, we believe that a significant factor that contributed in the management outcome of this case was the early-stage detection of the tumor, despite previous imaging studies showing a renal mass. Furthermore, we hold the perspective that the patient's presentation during the first trimester afforded the opportunity to judiciously formulate the management strategy.

In accordance with current guidelines, the preferred initial imaging modality for pregnant patients should generally not involve the use of a CT scan, except in cases where urgent diagnostic information is deemed essential. Given the efficacy of ultrasound and magnetic resonance imaging (MRI) in pregnant individuals, CT scans should be reserved for emergency situations or instances when MRI is not readily available [7]. However, contrast-enhanced CT scan continues to be the preferred imaging modality for diagnosing and staging renal tumors [2]. We maintain the perspective that this was the situation in our patient's diagnostic process, given the restricted accessibility of MRI in most public hospitals in Mexico. Thankfully, it seems that the exposure to ionizing radiation from the CT scan did not result in any adverse effects on the newborn.

Historically, pregnancy was regarded as an absolute contraindication to surgical intervention, primarily due to concerns regarding the risk of preterm labor and miscarriage. In addition, previous studies have revealed that women who underwent non-obstetric open surgical procedures while pregnant gave birth to infants with reduced birth weights, which carried an increased mortality rate during the first week following birth [8]. This notion has undergone a profound transformation owing to the advent of laparoscopy, because laparoscopic surgery has gained prominence across multiple surgical domains. Its primary benefits encompass decreased postoperative morbidity, diminished pain necessitating fewer analgesics, a faster restoration of bowel function, shortened hospitalization, and, consequently, a quicker return to usual activity levels. These benefits collectively hold significant value for pregnant patients [9].

Nevertheless, conducting laparoscopic procedures during pregnancy entails inherent risks. There exists a potential for injury to the gravid uterus. The occurrence of vena cava compression, attributed to heightened insufflation pressures and table positioning, can result in decreased maternal cardiac output, subsequently inducing fetal hypotension and hypoxia. The absorption of CO_2_ utilized for insufflation can give rise to fetal acidosis. Moreover, the pneumoperitoneum possesses the potential to exert pressure on blood vessels, resulting in a decrease in the uterine blood flow. In addition, performing laparoscopy can introduce heightened technical complexities during pregnancy. Nevertheless, recent literature underscores laparoscopic radical nephrectomy as a favored approach for management during pregnancy, especially when deemed feasible [8,10].

Managing renal masses during pregnancy presents a multifaceted challenge. There is a lack of consensus regarding the optimal timing of interventions and appropriate patient positioning during surgery. It is crucial to approach all solid renal tumors during pregnancy as potential RCC until proven otherwise. To mitigate this possibility, prompt nephrectomy, whether partial or radical, should be undertaken to establish a definitive diagnosis [2].

In instances where surgical intervention is deemed necessary during pregnancy, a collaborative approach involving obstetricians and anesthesiologists becomes essential. In terms of anesthesia, meticulous monitoring is vital to avert hypoxia, hypotension, and the use of nonsteroidal anti-inflammatory drugs. It is important to highlight that prolonged hypoxia and hypotension can lead to fetal demise. On the obstetric front, proactive preparations should be in place for potential emergent fetal delivery, depending on the outcome of the surgical procedure [11].

## Conclusions

This case underscores the unique characteristics and intricacies of managing RCC in pregnant patients. It presents an exceptional situation where both the patient's condition and fetal safety must be carefully considered. While the classic symptoms of this tumor strongly suggest its diagnosis, our case highlights the importance of prenatal counseling, as the incidental finding of a renal tumor during a screening ultrasound is not an uncommon form of diagnosis. Given the absence of standardized diagnostic or treatment criteria, a multidisciplinary approach is essential for optimal management, and each case should be revised individually. Our case report advocates for laparoscopic radical nephrectomy as surgical treatment whenever feasible and when the required equipment is available, based on the results obtained in this patient. Nonetheless, we also acknowledge that the use of ionizing radiation in a pregnant patient is a weakness of this case. Nevertheless, our outcomes were successful for both the patient, who remains disease-free, and fetal development, which progressed optimally.
